# Electron-lattice energy relaxation in laser-excited thin-film Au-insulator heterostructures studied by ultrafast MeV electron diffraction

**DOI:** 10.1063/1.4995258

**Published:** 2017-07-21

**Authors:** K. Sokolowski-Tinten, X. Shen, Q. Zheng, T. Chase, R. Coffee, M. Jerman, R. K. Li, M. Ligges, I. Makasyuk, M. Mo, A. H. Reid, B. Rethfeld, T. Vecchione, S. P. Weathersby, H. A. Dürr, X. J. Wang

**Affiliations:** 1 Faculty of Physics and Centre for Nanointegration Duisburg-Essen, University of Duisburg-Essen, Lotharstrasse 1, 47048 Duisburg, Germany; 2 SLAC National Accelerator Laboratory, 2575 Sand Hill Rd., Menlo Park, California 94025, USA; 3 School of Materials and Engineering, Shanghai Jiao Tong University, 800 Dongchuan Road, Shanghai, China; 4 Department of Physics and OPTIMAS Research Center, Technical University Kaiserslautern, Erwin-Schrödinger-Strae 46, 67663 Kaiserslautern, Germany

## Abstract

We apply time-resolved MeV electron diffraction to study the electron-lattice energy relaxation in thin film Au-insulator heterostructures. Through precise measurements of the transient Debye-Waller-factor, the mean-square atomic displacement is directly determined, which allows to quantitatively follow the temporal evolution of the lattice temperature after short pulse laser excitation. Data obtained over an extended range of laser fluences reveal an increased relaxation rate when the film thickness is reduced or the Au-film is capped with an additional insulator top-layer. This behavior is attributed to a cross-interfacial coupling of excited electrons in the Au film to phonons in the adjacent insulator layer(s). Analysis of the data using the two-temperature-model taking explicitly into account the additional energy loss at the interface(s) allows to deduce the relative strength of the two relaxation channels.

Modern electronic devices represent complex 3-dimensional heterostructures with nano-scale dimensions where the high current densities pose severe challenges for the thermal device design.^1^ Therefore, an improved understanding of the fundamental interactions that determine non-equilibrium energy relaxation and dissipation in such systems is of major importance. Electron-phonon coupling,^2^ in particular, determines the electronic transport properties and is also responsible for heat generation through the transfer of electronic excess energy to the lattice.

Despite numerous studies, it is still an open and controversially discussed question to which extent and by which mechanisms electron-phonon coupling is affected in nano-scale materials. For thin films, some studies report a thickness independent behavior,^3,4^ while others find an increase of the electron-phonon coupling strength with decreasing film thickness.^5–7^ In the cited work, time-resolved optical techniques were applied, which probe predominantly electronic properties, but only very indirectly the lattice degrees of freedom. Moreover, interpretation of measured optical transients in terms of electronic relaxation processes is not straight-forward since it requires a detailed understanding of the optical response under the strongly non-equilibrium conditions generated by short-pulse laser excitation.^8–11^

In contrast, time-resolved diffraction techniques employing ultrafast electron- or X-ray pulses provide direct structural sensitivity and find increasing use also for the investigation of energy relaxation processes in laser-excited materials including Au,^12–17^ the material studied in this work. However, systematic diffraction studies, which addressed explicitly the size dependence of electron-phonon coupling or the role of interfaces in nano-scale material systems, are almost missing.

Only recently we have applied time-resolved electron diffraction with MeV electron pulses to study the thickness dependence of the electron-lattice energy relaxation in thin Bi-films.^18^ Our experiments revealed an increased relaxation speed with decreasing film thickness indicating direct coupling of metal electrons to phonons of the insulating substrate,^19,20^ a process believed to be particularly effective as long as electrons and lattice are not in equilibrium.^6,21,22^ However, in Bi, different mechanisms—phonon softening^23–25^ and squeezing^26^ as well as normal electron-phonon coupling—contribute to the incoherent lattice response making a direct determination of the lattice temperature evolution difficult.

In this work, we extend, therefore, our time-resolved diffraction studies to a simpler material system, namely, thin film Au-insulator heterostructures. Through precise measurements of the transient Debye-Waller effect over an extended excitation range and for different sample configurations with respect to thickness and number of interfaces, we are able to separate the different contributions governing energy relaxation and to directly follow the transient increase of the r.m.s. atomic displacement. We find an accelerated relaxation when the film thickness is reduced or the Au-film is capped with an insulator top-layer, thus providing an additional Au-insulator interface. This represents strong support for the above mentioned cross-interfacial electron-phonon coupling scenario.

Experiments were carried out using the MeV Ultrafast Electron Diffraction (UED) facility recently established at SLAC National Accelerator Laboratory, which has been described in detail elsewhere.^27^ In brief, UED@SLAC consists of a S-band photocathode RF gun, which is driven by the frequency-tripled output of a precisely synchronized fs Ti:sapphire laser system and an ultra-stable klystron modulator. It provides ultrashort electron pulses at relativistic energies. The experiments reported here were carried out at a repetition rate of 120 Hz with pulses of approx. 2 × 10^5^ electrons per pulse, a bunch duration of $\tau_{\text{bunch}}\approx$ 250 fs FWHM, and a kinetic energy of $E_{\text{kin}}=3.7\;\text{MeV}$ (γ=8.2). These pulses were focused by a solenoid to a spot size of 200 *μ*m FWHM in the sample plane. Diffraction experiments were performed in normal incidence transmission geometry [see schematic in Fig. 1(a)], and the scattered electrons were recorded by a phosphor based single electron sensitive detector, which was placed 3.5 m away from the sample providing a momentum resolution of about 0.14 Å^−1^. The diffraction patterns were calibrated using an epitaxial Au-sample as a reference.^27^ This calibration revealed a strictly linear relation between the scattering angle and the length of the reciprocal lattice vector of the corresponding Bragg-reflections. Moreover, doubling the bunch charge through an increase of the UV laser power driving the photo-gun did not impair the momentum resolution. From both we conclude that space charge induced aberrations and trajectory displacement effects are negligible for the given experimental conditions.

**FIG. 1.**
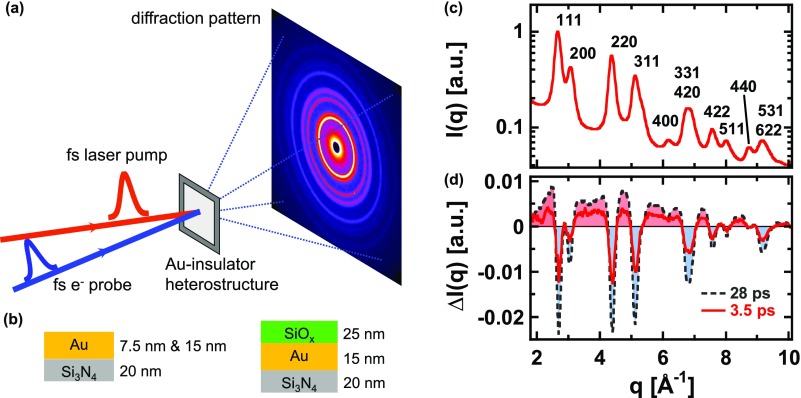
(a) Schematic of the experimental geometry. Thin film Au-insulator heterostructures are irradiated with 60 fs, 400 nm laser pulses (near normal incidence). Their structural response is probed by diffraction of 250 fs, 3.7 MeV kinetic energy electron pulses in normal incidence transmission geometry. (b) Sample layout. (c) Scattering intensity *I*(*q*) as a function of momentum transfer *q* of a 15 nm Au film on 20 nm Si_3_N_4_. (d) Difference scattering pattern $\Delta I\left(q,\Delta t\right)=I\left(q,\Delta t\right)-I_{0}\left(q\right)$ ($I_{0}\left(q\right)$: unpumped) for delay times Δt = 3.5 ps (red) and Δt = 28 ps (black).

For time-resolved measurements, 400 nm laser pulses with a duration of about 60 fs were obtained by second harmonic generation from the same Ti:sapphire laser and used for sample excitation. They were focused to a spot size of about 420 *μ*m FWHM (with an approximately Gaussian intensity distribution and an energy stability of about 1%) at an angle of incidence of 3°. The spot sizes of both, the electron probe pulses and the laser pump pulses as well as their spatial overlap were regularly monitored by mounting a YAG-scintillator exactly in the sample plane on a motorized stage and observing the fluorescence with an imaging CCD.

As shown schematically in Fig. 1(b), samples comprised polycrystalline thin films of Au with 7.5 nm and 15 nm thickness, respectively, deposited on 20 nm, free standing, amorphous ${\text{Si}}_{3}N_{4}$ membranes supported by a Si wafer frame using an anodic vacuum arc^28^ operated under high vacuum. 15 nm Au-films on 20 nm ${\text{Si}}_{3}N_{4}$ with an additional 25 nm amorphous SiO_x_ (with x close to 2) top-layer have been prepared with the same deposition technique.

As an example, the diffraction signal *I*(*q*) of a non-excited 15 nm Au film on Si_3_N_4_ (without SiO_x_ top-layer) as a function of momentum transfer q≈2π/λ·θ (λ=0.003 Å: De Broglie wavelength, *θ*: Scattering angle) obtained by azimuthal integration (along lines of constant *q*) of the recorded scattering image is shown in Fig. 1(c). Since the Si_3_N_4_ substrate (as well as the SiO_x_ in the sample with the additional top-layer) is amorphous and because of the low Z, it makes only a weak contribution to the scattering background. Upon pumping the sample, the diffraction intensity changes as can be seen in Fig. 1(d) showing transient difference scattering pattern pumped–unpumped for time delays Δt of 3.5 ps (red) and 28 ps (black-dashed), respectively, for the same film after excitation at an incident fluence of *F* = 1.3 mJ/cm^2^ (all fluence values quoted here refer to the incident peak fluence of the nearly Gaussian fluence distribution of the focused pump beam). A decrease of the Bragg-peak intensities as well as an increase of the diffuse background in between can be recognized. As will be discussed in detail below, both features can be attributed to the increase of the r.m.s. atomic displacement in the Au film after sample excitation. It should be noted that due to the normal-incidence geometry and the very short de Broglie wavelength (i.e., extremely flat Ewald-sphere) our experiment is only sensitive to atomic motion in the film plane and thus not affected, for example, by the excitation of longitudinal strain waves,^13,15,29^ which develop on *acoustic* time-scales *d*/*c* (*d*: film thickness, *c*: speed of sound).

To quantitatively analyze the transient diffraction data, the integrated signal of those Bragg-peaks, which are either sufficiently strong or well separated from other peaks, has been determined by fitting them separately with a Gaussian function superimposed on a (linear) background for each pump-probe time delay Δt. Figure 2(a) shows as an example the result of this analysis for the same 15 nm Au-film and the same pump fluence of *F* = 1.3 mJ/cm^2^ as in Fig. 1(c). The diffraction signal has been normalized to the value measured at negative delay times, i.e., before sample excitation. As violet data points [Fig. 2(a)] also depicts the time dependence of the diffuse scattering signal measured between the (200)- and (220)-reflection from 3.5 Å^−1^ to 3.9 Å^−1^.

**FIG. 2.**
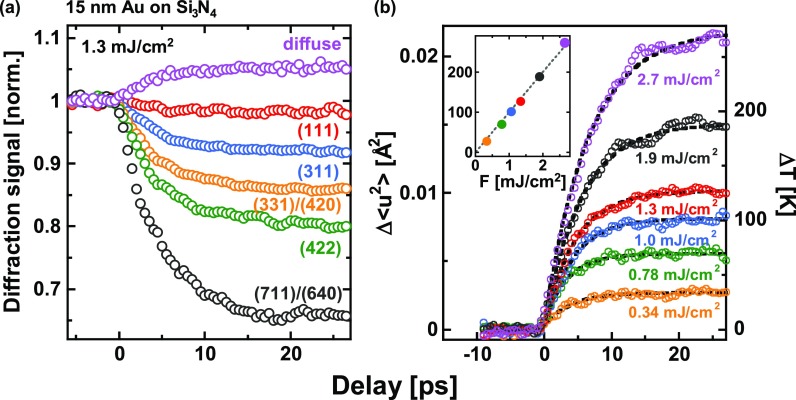
Transient diffraction data for a 15 nm Au film on 20 nm Si_3_N_4_. (a) Normalized integrated diffraction efficiency of various Bragg-peaks (*hkl*) as a function of pump-probe time delay for an excitation fluence F = 1.3 mJ/cm^2^; the violet data points show the time dependence of the diffuse scattering signal measured at *q* = (3.7 ± 0.2) Å^−1^. (b) Change of the r.m.s. displacement $\Delta 〈u^{2}〉\left(\Delta t\right)$ as a function of pump-probe time delay for various excitation fluences. The right ordinate represents the derived lattice temperature (see text); the black dashed curves are fits to the data with exponential time-dependencies [see Eq. (2)]. The inset shows the maximum laser-induced temperature rise $\Delta T_{\infty }$ as a function of pump fluence.

The integrated diffraction signal of the different Bragg-peaks exhibits an order-dependent decrease (Debye-Waller effect) within a few ps, while the diffuse scattering increases on a similar time-scale. As mentioned earlier, this can be attributed to the laser-induced increase of the r.m.s. atomic displacement. For further analysis, we use the logarithmic form of the Debye-Waller-factor

$$-\ln\left(\frac{I_{hkl}\left(\Delta t\right)}{I_{hkl}^{0}}\right)=\frac{1}{3}\Delta 〈u^{2}〉(\Delta t)\cdot G_{hkl}^{2}.$$ (1)

Herein, $I_{hkl}^{0}$ denotes the scattering signal of the unpumped sample (measured at negative time delays), *G_hkl_* the length of the reciprocal lattice vector corresponding to reflection (*hkl*), and $\Delta 〈u^{2}〉$ the transient change of the r.m.s. displacement upon laser excitation. It needs to be emphasized that all data follow (within the experimental accuracy) Eq. (1), i.e., for a given fluence and at a given delay time the negative logarithm of the normalized intensity exhibits a linear dependence on $G_{hkl}^{2}$, clearly indicating a completely incoherent lattice response. $\Delta 〈u^{2}〉$ can then be directly determined from the slope of these linear dependencies. Results for a set of different pump fluences, again for the same 15 nm Au-film as before, are shown in Fig. 2(b).

Opposite to our previous work on thin Bi-films,^18^ where electronic excitation leads to phonon softening^23–25^ and, therefore, does not allow to obtain the lattice temperature as a function of time, such effects are not expected to occur in Au at the excitation levels of our experiments.^30,31^ We used published data on the temperature dependence of the Debye-Waller-factor of Au^32,33^ to convert the experimental $\Delta 〈u^{2}〉\left(\Delta t\right)$ into the transient temperature rise ΔT(Δt). The result of this conversion is shown in Fig. 2(b) at the right ordinate. The inset shows the maximum laser-induced temperature rise $\Delta T_{\infty }$ as a function of pump fluence, which exhibits a linear increase with a slope of $\beta =(100\pm 2)\;K/\left({\text{mJ}/\text{cm}}^{2}\right)$.

The black-dashed lines in Fig. 2(b) represent fits to the experimental data with exponential time-dependencies

$$\Delta T\left(\Delta t\right)=\Delta T_{\infty }\cdot \left(1-e^{-\Delta t/\tau }\right),$$ (2)

which allows to derive the corresponding electron-lattice relaxation time *τ* as a function of pump fluence. Similar measurements on 7.5 nm thick Au-films as well as 15 nm Au-films with the additional SiO_x_ top-layer have been analyzed in the same way. These results are summarized in Fig. 3(a), which shows as open circles for the three different sample configurations the experimentally determined relaxation time *τ* as a function of the laser-induced temperature rise $\Delta T_{\infty }$. These data are complemented by results shown in Fig. 3(b), which compares for the same final temperature rise $\Delta T_{\infty }\approx 275\;K$ the temporal evolution of ΔT in a 15 nm film with (blue) and without (red) the SiO_x_ top-layer.

**FIG. 3.**
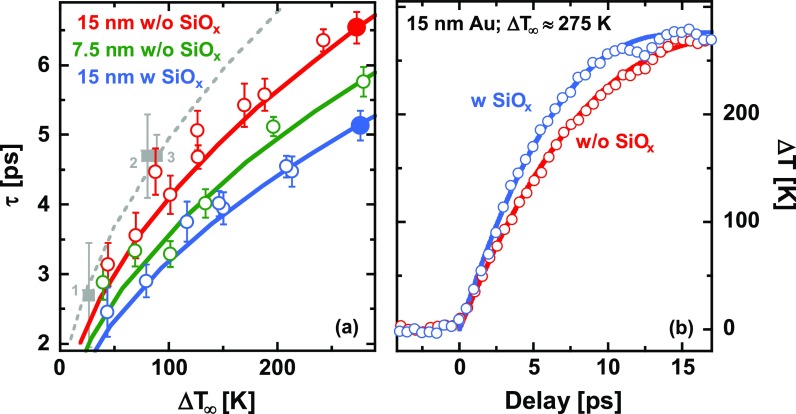
(a) Equilibration time *τ* for different Au-insulator heterostructures (blue: 25 nm SiO_*x*_ on 15 nm Au on 20 nm Si_3_N_4_; green: 7.5 nm Au on 20 nm Si_3_N_4_; red: 15 nm Au on 20 nm Si_3_N_4_) as a function of the asymptotic laser-induced temperature rise $\Delta T_{\infty }$. Results from the literature are included as grey squares: 1—Nakamura *et al.*,^17^ 2—Ligges *et al.*,^12^ 3—Chase *et al.*^16^ (b) ΔT(Δt) as a function of pump-probe time delay for a 15 nm Au film on 20 nm Si_3_N_4_ without (red data points) and with (blue data points) an additional SiO_x_ top layer (25 nm thickness). For both cases, a similar temperature increase $\Delta T_{\infty }\approx 275\;K$ is reached at long delay times; the corresponding data points are marked by full circles in (a). The solid and the grey-dashed curves in (a) and (b) represent results of calculations based on the two-temperature-model (TTM) as discussed in the text.

We would like to emphasize that using $\Delta T_{\infty }$ as a reference for comparing the different sample configurations represents a crucial point in our analysis of the relaxation behavior. This essentially takes out effects which result from differences in the absorption properties of the different thin-film structures. It also compensates for any experimental uncertainties as a consequence of slight day-to-day changes in the pump laser profile and misadjustment or drifts of the spatial overlap.

For all samples, the relaxation time increases as a function of $\Delta T_{\infty }$. However, for the same final temperature, relaxation is slowest for the bare 15 nm film on Si_3_N_4_, while it is faster for the thinner, 7.5 nm film as well as for the 15 nm film with the SiO_x_ top-layer. Since the material microstructure can influence the relaxation behavior,^34^ it is important to emphasize that all samples are prepared in the same way. Therefore, the data shown in Fig. 3 give clear evidence for a dependence of the relaxation rate on film thickness as well as on the number of Au-insulator interfaces. These observations are in agreement with our recent results on thin Bi-films^18^ as well as with some of the time-resolved optical studies,^5–7^ providing strong support for the concept of a cross-interfacial coupling of hot electrons in the Au-film to interface vibrational modes,^19,20^ which for metal-insulator interfaces mainly reside in the substrate.

To quantitatively analyze our results and to estimate the strength of both relaxation channels, we apply the well-known two-temperature model^35^ (TTM), which describes the response of the material by two coupled heat diffusion equations for the electronic and lattice system, respectively. Taking into account the small thickness of the films used in this experiment, which is comparable to the optical absorption depth [16 nm at the excitation wavelength of 400 nm (Ref. 36)] and much smaller than the ballistic range of excited electrons in Au of about 100 nm,^37,38^ we can assume a spatially homogeneous deposition of the optical energy into the electronic system of the Au-film. To account for the interface-mediated electron-phonon coupling, an additional loss term is introduced in the electron equation^5,6^

$${\dot{Q}}_{e}=-\sigma_{e}(T_{e}-T_{I}),$$ (3)

*σ_e_* represents the appropriate boundary conductance, and *T_e_* and *T_I_* the electron temperature in the Au-film and the relevant interface temperature, respectively. For the latter, we assume that it is identical to the lattice temperature in the film *T_L_*.^39^ Due to the short pump pulse duration and the few ps time-scale of interest, an instantaneous and homogeneous increase of the electronic temperature was chosen as starting condition (instead of an explicit laser excitation source term). We verified for a few selected cases with explicit space-dependent TTM-calculations that the temperature in the Au-films remains homogeneous (maximum variations over the film thickness are less than 1%) for all times even with the additional interface coupling term, resulting in the following equations, which are only time-dependent:

$$\begin{array}{c} c_{e}\;\frac{\partial T_{e}}{\partial t}=-\left(g+\frac{\sigma_{e}}{d}\right)\;\left(T_{e}-T_{L}\right)\\ c_{L}\;\frac{\partial T_{L}}{\partial t}=+g\;\left(T_{e}-T_{L}\right). \end{array}$$ (4)

Herein, $c_{e}=67.6\;J/\left(m^{3}\;K^{2}\right)\cdot T_{e}$ and $c_{L}=2.5\;\text{MJ}/\left(m^{3}\;K\right)$ denote the electronic and lattice specific heat, respectively. Equation (4) shows that under the assumption of a homogeneous temperature distribution the loss of electronic energy at the interface can be cast into an effective coupling parameter $g_{I}=\sigma_{e}/d$. The bulk electron-phonon coupling parameter *g* as well as the electronic boundary conductance *σ_e_* are treated as free parameters in a consistent manner, i.e., same *g* and identical $\sigma_{e,\;\text{sin}\;}$ of the Au-Si_3_N_4_-interface for all three samples.

The solid curves in Figs. 3(a) and 3(b) represent results of such calculations with $g=1.7\times 10^{16}\;W/\left(m^{3}\;K\right),\;\sigma_{e,\;\text{SiN}\;}=97\;\text{MW}/\left(m^{2}\;K\right)$, and $\sigma_{e,SiO}=197\;\text{MW}/\left(m^{2}\;K\right)$, which provide a good description for the whole set of experimental data [i.e., Fig. 3(a)]. Within the TTM, the increase of the relaxation time with temperature, which is observed for all sample configurations, can be attributed to the temperature dependence of the electronic specific heat. Moreover, we have to conclude that the effective interface contribution *g_I_* is comparable to the electron-phonon coupling strength *g* in the bulk for both, the 7.5 nm Au-film on Si_3_N_4_ ($g_{I}=1.3\times 10^{16}\;W/\left(m^{3}\;K\right)$) and the 15 nm Au-film on Si_3_N_4_ with the SiO_x_ top-layer ($g_{I}=1.8\times 10^{16}\;W/\left(m^{3}\;K\right)$). While there is some ambiguity in the relative strength of the bulk and interface contribution (i.e., a slightly reduced *g* and correspondingly increased values for the *σ_e_* provide a similar good description), TTM-calculations with an electron-phonon coupling parameter $g=1.7\times 10^{16}\;W/\left(m^{3}\;K\right)$ [grey-dashed curve in Fig. 3(a)] are also in good agreement with published data on free-standing Au-films^12,16,17^ [grey squares in Fig. 3(a)], where the relaxation is not affected by any cross-interfacial coupling.

It needs to be emphasized that for a given $\Delta T_{\infty }$ due to the additional loss of energy to the insulating layer(s), the energy density that needs to be deposited and thus the peak electronic temperature increases the larger *g_I_* gets. Within the TTM the temperature rise $\Delta T_{\infty }$ of a film with absorption *A* and thickness *d* as a function of the incident laser fluence $F_{\text{inc}}$ can be estimated as

$$\Delta T_{\infty }=\frac{A}{d}\cdot \frac{g}{g+\frac{\sigma_{e}}{d}}\cdot \frac{F_{inc}}{c_{L}}.$$ (5)

The absorption at 400 nm of the 7.5 nm and the 15 nm film without SiO_x_ top-layer has been measured independently as A=(0.28±0.03) and A=(0.50±0.05), respectively (unfortunately, we were not able to measure the absorption of the 15 nm film with SiO_x_ top-layer since it got damaged during the UED measurements). With these values, Eq. (5) yields a differential temperature rise $\beta =d\Delta T_{\infty }/dF_{inc}$ of $(85\pm 9)\;K/\left({\text{mJ}/\text{cm}}^{2}\right)$ and $(96\pm 10)\;K/\left({\text{mJ}/\text{cm}}^{2}\right)$ for 7.5 nm and 15 nm film thickness, respectively, in reasonable agreement with the experimental values of $(102\pm 3)\;K/\left({\text{mJ}/\text{cm}}^{2}\right)$ (7.5 nm) and $(100\pm 2)\;K/\left({\text{mJ}/\text{cm}}^{2}\right)$ [15 nm; see also the inset in Fig. 2(b)]. This further supports our conclusion on the relevance of the cross-interfacial coupling in explaining the observed relaxation behavior.

Finally, comparing our data as well as the above mentioned results,^12,16,17^ it is noticeable that the electron-phonon coupling parameter derived from time-resolved diffraction data seems to be smaller than the values usually deduced in time-resolved optical studies (e.g., Ref. 3 and references therein), which monitor (although indirectly) the electron dynamics. In contrast, time-resolved diffraction probes directly the response of the lattice by measuring the r.m.s. atomic displacements. It has been shown recently that even in simple metals the phonon system is not in thermal equilibrium during the electron-lattice equilibration,^16,40^ and hot electrons interact predominantly with higher frequency phonons. Therefore, conversion of the measured $\Delta 〈u^{2}〉$ into a lattice temperature using the equilibrium Debye-Waller factors^32,33^ might initially underestimate the transient energy transfer to the lattice. However, this does not affect our conclusions on the importance of interface-effects for the speed of the relaxation process.

In summary, time-resolved diffraction with femtosecond, relativistic electron pulses has been used to study electron-lattice equilibration in thin film Au-insulator heterostructures after ultrafast laser excitation. Our data reveal a striking dependence of the relaxation rate on film thickness as well as on the presence of an additional Au-insulator interface, which is taken as evidence for a cross-interfacial electron-phonon coupling process. Calculations based on a modified two-temperature-model allow to quantitatively estimate the strength of bulk and interface contributions which are found to be comparable for the studied systems. Our results demonstrate the unique possibilities time-resolved diffraction techniques offer for the direct study of energy relaxation and dissipation in nano-scale material systems under highly non-equilibrium conditions.
